# Scientometric Analysis: University Hospital Versus University College of Medicine

**DOI:** 10.7759/cureus.12096

**Published:** 2020-12-15

**Authors:** Abraar Muneem, David R Hallan, Sage Gee, Sathvik Saineni, Usman Asad, Surav M Sakya

**Affiliations:** 1 Medicine, Penn State College of Medicine, Hershey, USA; 2 Neurosurgery, Penn State College of Medicine, Milton S. Hershey Medical Center, Hershey, USA; 3 Internal Medicine, Penn State College of Medicine, Milton S. Hershey Medical Center, Hershey, USA; 4 Medicine, Gandhi Medical College, Hyderabad, IND; 5 Dermatology, St. Anthony Hospital, Oklahoma City, USA

**Keywords:** scientometric, penn state, medicine, hershey, medical, center

## Abstract

Many medical specialties use scientometrics to assess the impact of publications, journals, and authors. The aim of this study was to analyze and compare trends of publications from a hospital medical center to publications from a college of medicine connected to that hospital and compare collaboration rates between them to other domestic and international institutions. We used Elsevier’s SCOPUS database to compare Penn State College of Medicine (PSCOM) publications to Hershey Medical Center (HMC) publications, analyzing 31,856 total publications. We hypothesized that HMC and PSCOM have room to improve on both internal and international collaborations. Our results show that despite PSCOM’s international collaboration being nearly three times higher than HMC, overall international collaboration is less than 2%, far below the US national average.

## Introduction

Scientometrics measures and analyzes scientific literature and is a subset of bibliometrics. Many medical specialties use bibliometrics to compile, use, and review the most-cited works [1]. This is especially useful, as the exponential rise in publications and resources makes it difficult for learners to process information efficiently. It identifies salient topics and assesses the impact of publications, journals, and authors. Furthermore, bibliometrics takes a snapshot in time of objective metrics, which can highlight scientific progression, historical trends, and prolific individuals.

The aim of this study was to identify historical trends in Penn State College of Medicine (PSCOM) publications and compare them to the trends of Hershey Medical Center (HMC) publications, especially as it relates to collaboration. We hypothesize that given their physical, financial, and leadership connections, in addition to shared research aims, that the collaboration rate between PSCOM and HMC will be higher than with all other institutions combined, that their top authors and most cited articles will be the same and that they will have similar publication rates in journals. We also hypothesize that international collaboration for PSCOM and HMC will be higher than the national average because of both institutions' focus on global health rotations and research projects.

## Materials and methods

A comprehensive search within Elsevier's SCOPUS was performed on May 2, 2020. Institution search was performed using the Boolean query "Penn State College of Medicine" and variations yielding Affiliation ID 60027671. Another search was performed using the Boolean query "Milton S. Hershey Medical Center" and variations yielding Affiliation ID 60013671. Data were collected, sorted, and analyzed by topic, affiliation, journal name, highest citations, authors' publications, and H-index. The Penn State Cancer Institute was considered a separate institution. All collaborating affiliations were obtained for both HMC and PSU. Calculated international collaboration rates in percentage were obtained by dividing all publications from international institutions by the total number of publications.

## Results

PSCOM has 18,327 total publications by 4,535 authors, and HMC has 16,259 total publications by 4,135 authors.

Research topics

**Figure 1:**
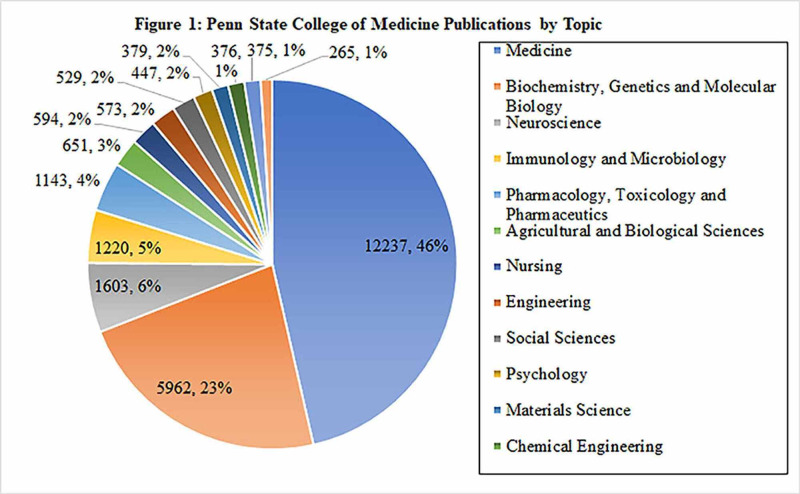
Penn State College of Medicine (PSCOM) publications by topic A total of 13 topics were included. The top three topics were Medicine (46%), Biochemistry, Genetics, and Molecular Biology (23%), and Neuroscience (6%). The bottom three topics were Chemical Engineering (1%), Multidisciplinary (1%), and Health Professions (1%).

**Figure 2:**
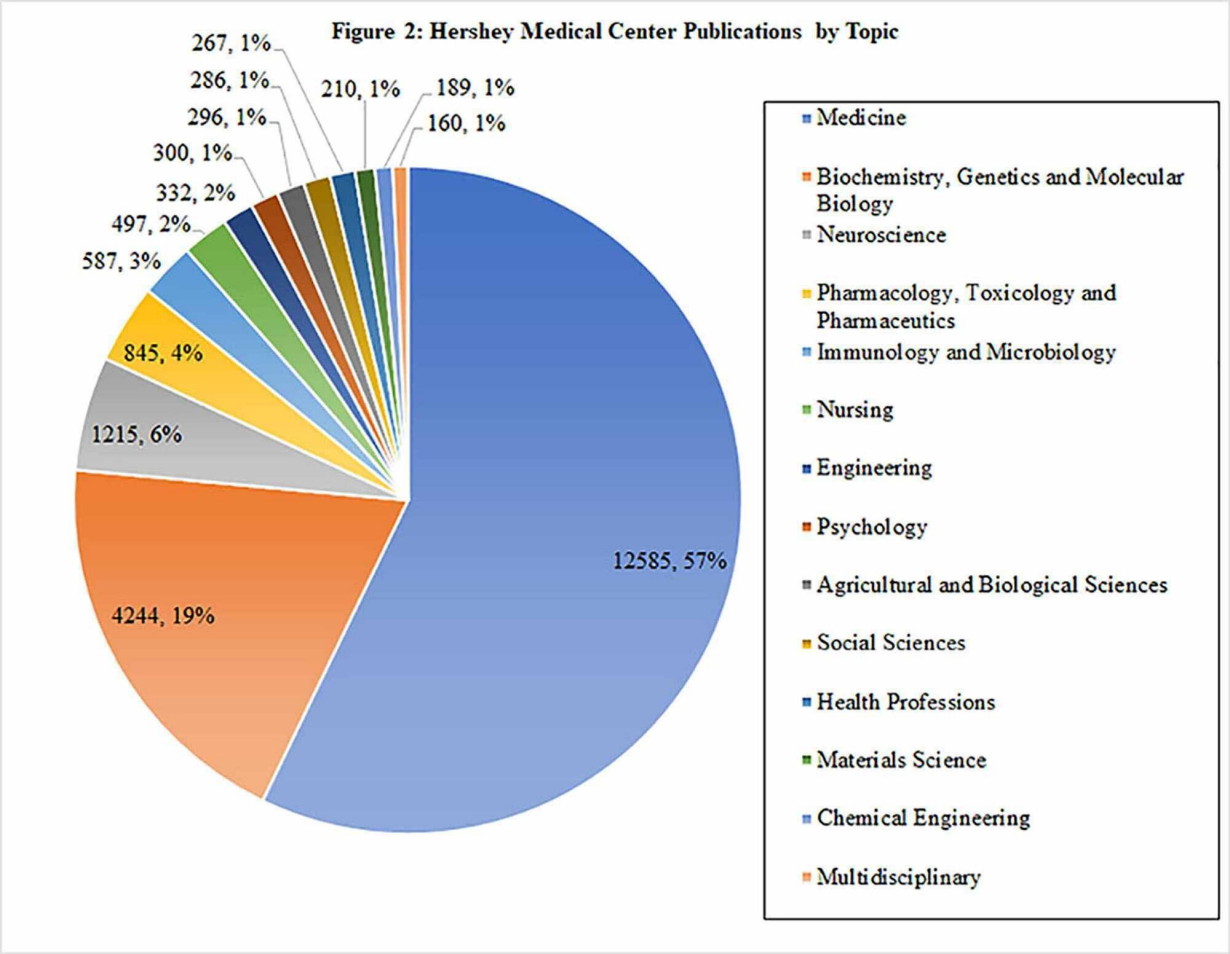
Hershey Medical Center (HMC) publications by topic A total of 13 topics were included. The top three topics were Medicine (57%, respectively), Biochemistry, Genetics and Molecular Biology (19%), and Neuroscience (6%). The bottom three topics were Materials Science (1%), Chemical Engineering (1%), and Multidisciplinary (1%).

For both PSCOM and HMC, the top three publication topics are Medicine (46% and 57%, respectively), Biochemistry, Genetics and Molecular Biology (23% and 19%, respectively), and Neuroscience (6% and 6%, respectively). The bottom three topics for PSCOM were Chemical Engineering (1%), Multidisciplinary (1%) and Health Professions (1%), whereas the bottom three topics for HMC were Materials Science (1%), Chemical Engineering (1%), and Multidisciplinary (1%) (Figures 1-2).

Journals

**Figure 3:**
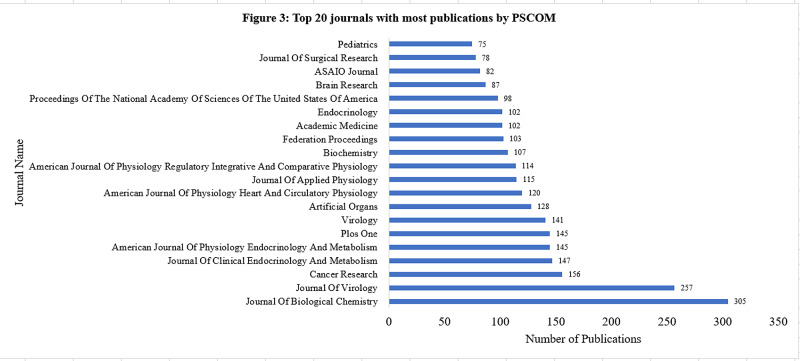
Top 20 journals with most publications by PSCOM The top three journals were Journal of Biological Chemistry (305), Journal of Virology (257), and Cancer Research (156). PSCOM: Penn State College of Medicine

**Figure 4:**
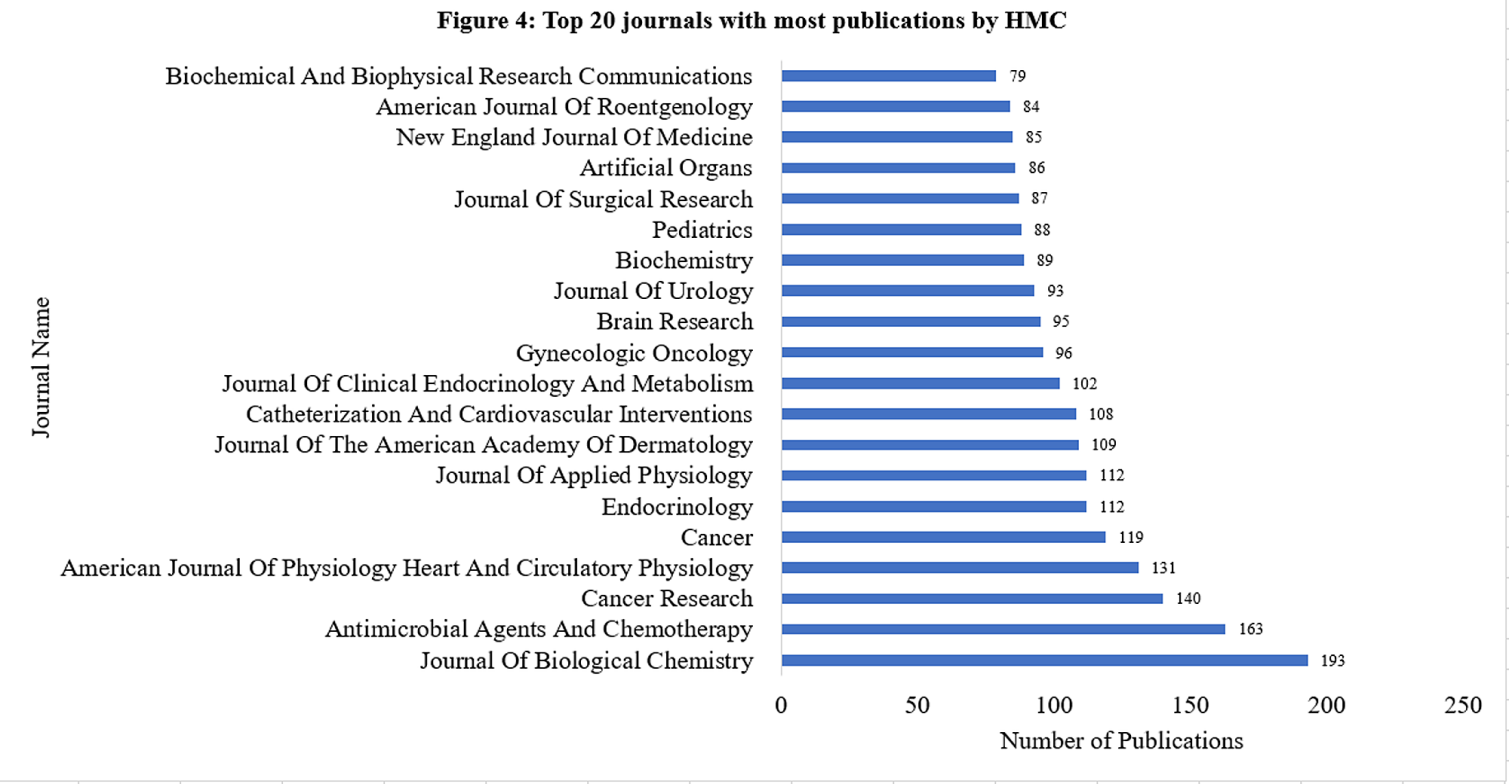
Top 20 journals with most publications by HMC The top three journals were Journal of Biological Chemistry (193), Antimicrobial Agents and Chemotherapy (163), and Cancer Research (140). HMC: Hershey Medical Center

The top three journals in which PSCOM published were the Journal of Biological Chemistry (305 publications), Journal of Virology (257), and Cancer Research (156). The top three journals in which HMC chose to publish were the Journal of Biological Chemistry (193), Antimicrobial Agents and Chemotherapy (163), and Cancer Research (140) (Figures 3-4).

Collaboration and affiliation

Both PSCOM and HMC had one another as their top collaborator at 5,659 publications (Table 1). They also had Penn State University as their second top collaborator, with 2,080 and 1,204 collaborations, respectively. PSCOM's third most common affiliation was with the University of Pennsylvania (397 publications), followed by Harvard Medical School (361). HMC's third most common collaborator was Harvard Medical School (259), followed by the University of Pennsylvania (235).

**Table 1:** Number of publications by top 20 collaborating affiliations Both PSCOM and HMC had one another as their top collaborator in 5,659 publications. They also had Penn State University as their second top collaborator, with 2,080 and 1,204 collaborations, respectively. PSCOM's third most common affiliation was with the University of Pennsylvania (397 publications), followed by Harvard Medical School (361). HMC's third most common collaborator was Harvard Medical School (259), followed by the University of Pennsylvania (235). PSCOM: Penn State College of Medicine; HMC: Hershey Medical Center

Number of Publications					
Penn State College of Medicine			Hershey Medical Center		
Affiliation Name	# of Publications	%	Affiliation Name	# of Publications	%
Penn State Health Milton S. Hershey Medical Center	5,659	47.3%	Penn State College of Medicine	5,659	56.5%
Pennsylvania State University	2,080	17.4%	Pennsylvania State University	1,204	12.0%
University of Pennsylvania	397	3.3%	Harvard Medical School	259	2.6%
Harvard Medical School	361	3.0%	University of Pennsylvania	235	2.3%
Penn State University	344	2.9%	VA Medical Center	219	2.2%
National Institutes of Health, Bethesda	324	2.7%	UT Southwestern Medical Center	219	2.2%
The University of North Carolina at Chapel Hill	258	2.2%	Case Western Reserve University	205	2.0%
University of California, San Francisco	256	2.1%	Penn State University	203	2.0%
VA Medical Center	242	2.0%	Massachusetts General Hospital	183	1.8%
University of Michigan, Ann Arbor	233	1.9%	National Institutes of Health, Bethesda	175	1.7%
University of Pennsylvania School of Medicine	223	1.9%	University of Texas MD Anderson Cancer Center	161	1.6%
University of Pittsburgh	208	1.7%	University of California, San Francisco	159	1.6%
Johns Hopkins University	193	1.6%	Cleveland Clinic Foundation	158	1.6%
National Cancer Institute	179	1.5%	University of Michigan, Ann Arbor	154	1.5%
University of California, Los Angeles	175	1.5%	Mayo Clinic	147	1.5%
Brigham and Women's Hospital	172	1.4%	Brigham and Women's Hospital	140	1.4%
Yale School of Medicine	170	1.4%	Duke University Medical Center	139	1.4%
University of Texas MD Anderson Cancer Center	162	1.4%	University of Washington, Seattle	136	1.4%
The University of Alabama at Birmingham	159	1.3%	University of Pennsylvania School of Medicine	134	1.3%
Columbia University in the City of New York	159	1.3%	National Cancer Institute	133	1.3%
Total	11,954	100.0%	Total	10,022	100.0%

International collaboration rates for both institutes are low [2]. The rate of international collaboration for HMC is 0.71% (115) and PSCOM is 1.88% (345).

Citations

The most cited paper for HMC is “Cardiac-Resynchronization Therapy With or Without an Implantable Defibrillator in Advanced Chronic Heart Failure” by Bristow et al. with 4,278 citations [3]. The most cited paper for PSCOM is “Guidelines for the Use and Interpretation of Assays for Monitoring Autophagy” by Klionisky et al. with 2,462 citations [4].

Authors and patterns of production

The most prolific author for HMC is Anthony E. Pegg with a total of 588 publications. H-index is a measure of an author’s publication productivity and the citation impact of the publication. Dr. Pegg also holds the highest H-index of 88, making him the most impactful and relevant author at HMC. The most prolific author for PSCOM is Vijay K. Varadan with a total of 715 publications. Leonard S. Jefferson holds the highest H-index of 75 at PSCOM (Table 2).

**Table 2:** Top 20 authors by the number of publications The most prolific author for HMC is Anthony E. Pegg with a total of 588 publications. H-index is a measure of an author’s publication productivity and citation impact of the publication. Dr. Pegg also holds the highest H-index of 88 making him the most impactful and relevant author at HMC. The most prolific author for PSCOM is Vijay K. Varadan with a total of 715 publications. Leonard S. Jefferson holds the highest H-index of 75 at PSCOM.

Authors by Number of Publications					
Penn State College of Medicine			Hershey Medical Center		
Author Name	Number of Publications	H-Index	Author Name	Number of Publications	H-Index
Varadan, Vijay K.	715	53	Pegg, Anthony E.	588	88
Amin, Shantu G.	448	59	Appelbaum, Peter Colin	419	67
Demers, Laurence M.	440	68	Lipton, Allan M.	388	77
Scott, Ingrid Ursula	417	68	Rapp, Fred D.	371	36
Legro, Richard S.	391	71	Connor, James R.	331	75
Wu, Rongling	381	39	Naccarelli, Gerald V.	272	46
Belani, Chandra P.	372	59	Mailman, Richard B.	259	51
Lang, Charles	368	63	Naeye, Richard L.	245	54
Chinchilli, Vernon M.	365	66	Schmitz, Kathryn H.	238	60
Zagon, lan S.	333	51	Sinoway, Lawrence I.	212	49
Jefferson, Leonard S.	315	75	Gilchrist, Ian C.	210	28
Lee, Peter Allen	314	54	Wu, Jang Yen	198	56
Vesell, Elliot S.	311	46	Ruffin Iv, Mack Thomas	196	41
Kimball, Scot R.	296	70	Wang, Kelin	195	55
Ündar, Akif	294	33	Graham, William P.	175	24
Raman, J. D.	290	50	Ehrlich, H. Paul	172	43
Pierce, William S.	287	31	Zaino, Richard J.	169	56
Dokholyan, Nikolay V.	285	58	Huang, Xuemei	167	39
Gelenberǵ, Alan J.	284	54	Hopper, Kenneth D.	161	38
Hollenbeak, Christopher S.	278	39	Kreider, John W.	157	33

## Discussion

The most notable finding from our data analysis is the low international collaboration rate in both institutions (HMC 0.71%, PSCOM 1.88%). In the 2020 Indicators Report published by the National Science Foundation, 39% of US peer-reviewed publications have international collaborators, with China being its closest collaborator, followed by the United Kingdom [2]. Previous studies have shown that collaboration with international researchers significantly improved the quality and quantity of research publications as judged by the impact factor of the journals where the works were published, especially in developing countries [5-6]. Dakik et al. 2006 reiterated this finding and found that their institution, the American University of Beirut, had a 9% international collaboration rate [7]. International collaborations have also been shown to increase the impact of research, as measured by citations [8]. Ranking 23rd of top national research institutions in expenditure, HMC and PSCOM have the potential to improve in international collaborations as well as in collaborations with institutes in developing countries [9].

In the process of improving international collaboration, some factors might explain the potential obstacle to reach a higher international collaboration rate. The Penn State system is a public institution with most of the research activity being federally funded, and it is subjected to federal government regulations for international research collaboration. The complicated approval process of the international component might hinder HMC and PSCOM's motivation for international collaboration [10].

PSCOM and HMC share an organic connection and geographical proximity. Yet, of their combined 31,856 publications, only 5,659 (17.7%) were collaborative works between these two institutions (p-value <0.00001). This is surprising given the graduate student workforce available to PSCOM, which consists of many MD and MD/PhD students who also have clinical duties at HMC, and the number of HMC physicians who hold professorship positions at PSCOM.

The most prolific authors from each institution are likewise dissimilar, as are the most cited works. Likewise, although the top journals that PSCOM and HMC publish in are similar, they are not the same and have different rates of publication (p-value < 0.00001). 

Overall, all of our null hypotheses are not rejected, as our analysis provides data contrary to our hypotheses that given their physical, financial, and leadership connections, in addition to shared research aims, that the collaboration rate between PSCOM and HMC will be higher than all other institutions combined, that their top authors and most cited articles will be the same, and that they will have similar publication rates in journals, as well as data contrary to international collaboration being high.

Our limitations include our search being limited to articles contained within Elsevier's database, which, although fairly comprehensive with 70,000 institutional profiles and 1.4 billion cited references, does not capture the entirety of everything ever published. In addition, though we report 5,659 collaborative papers, this number may be lower because one author may have affiliations at both PSCOM and HMC. This stresses the need for more collaboration.

## Conclusions

This study provides a snapshot in time of PSU and HMC publication statistics. Both institutions are producing high-quality research published in top national scientific journals. However, both institutions should make it their goal to foster greater international collaboration as well as collateral collaboration, as an average of 1.30% international collaboration is significantly lower than the US publication average and is not reflective of the Penn State system’s research potential. A 17.7% collaboration rate between sister institutions that share similar goals and climates is likewise something that can be improved upon. The authors suggest that this study be repeated for other sister institutions throughout the US to compare collaboration rates. This study should also be repeated every few years to note trends and measure efforts to improve collaboration.
